# The transformation of spinal curvature into spinal deformity: pathological processes and implications for treatment

**DOI:** 10.1186/1748-7161-1-3

**Published:** 2006-03-31

**Authors:** Martha C Hawes, Joseph P O'Brien

**Affiliations:** 1 Division of Plant Pathology and Microbiology, Department of Plant Sciences, University of Arizona, Tucson AZ 85721, USA; 2 National Scoliosis Foundation, 5 Cabot Place, Stoughton MA 02072, USA

## Abstract

**Background:**

This review summarizes what is known about the pathological processes (e.g. structural and functional changes), by which spinal curvatures develop and evolve into spinal deformities.

**Methods:**

Comprehensive review of articles (English language only) published on 'scoliosis,' whose content yielded data on the pathological changes associated with spinal curvatures. Medline, Science Citation Index and other searches yielded > 10,000 titles each of which was surveyed for content related to 'pathology' and related terms such as 'etiology,' 'inheritance,' 'pathomechanism,' 'signs and symptoms.' Additional resources included all books published on 'scoliosis' and available through the Arizona Health Sciences Library, Interlibrary Loan, or through direct contact with the authors or publishers.

**Results:**

A lateral curvature of the spine–'scoliosis'–can develop in association with postural imbalance due to genetic defects and injury as well as pain and scarring from trauma or surgery. Irrespective of the factor that triggers its appearance, a sustained postural imbalance can result, over time, in establishment of a state of continuous asymmetric loading relative to the spinal axis. Recent studies support the longstanding hypothesis that spinal deformity results directly from such postural imbalance, irrespective of the primary trigger, because the dynamics of growth within vertebrae are altered by continuous asymmetric mechanical loading. These data suggest that, as long as growth potential remains, evolution of a spinal curvature into a spinal deformity can be prevented by reversing the state of continuous asymmetric loading.

**Conclusion:**

Spinal curvatures can routinely be diagnosed in early stages, before pathological deformity of the vertebral elements is induced in response to asymmetric loading. Current clinical approaches involve 'watching and waiting' while mild reversible spinal curvatures develop into spinal deformities with potential to cause symptoms throughout life. Research to define patient-specific mechanics of spinal loading may allow quantification of a critical threshold at which curvature establishment and progression become inevitable, and thereby yield strategies to prevent development of spinal deformity.

*Even within the normal spine there is considerable flexibility with the possibility of producing many types of curves that can be altered during the course of normal movements. To create these curves during normal movement simply requires an imbalance of forces along the spine and, extending this concept a little further, a scoliotic curve is produced simply by a small but sustained imbalance of forces along the spine. In fact I would argue that no matter what you believe to be the cause of AIS, ultimately the problem can be reduced to the production of an imbalance of forces along the spine *[1].

## Introduction

The defining property of humans and other vertebrates is the vertebral column, housing as it does a multifaceted sensory-response system integrating every aspect of movement, form, and function. Therefore it is not surprising that a deformity of the spine can be associated with a diverse array of pathological consequences. The spinal functions and structures of scoliosis patients have been described and compared with those of control subjects in hundreds of research articles; a representative sample is provided in Table 1. The presence of scoliosis has been considered with regard to a possible relationship with factors including posture, balance, muscle structure, psychology, height, vision, hearing, hormones, birth injury, and genetics. To date, for the majority of scoliosis patients, issues of cause and effect remain unclear, in part because a deformed spine has potential to induce diverse secondary changes by virtue of its comprehensive role in human biology. Numerous hypotheses about why spinal deformities develop in certain individuals have been proposed in past decades [e.g 1–7], and a current synthesis of these concepts has been published [8]. In recent years, for the first time, progress has been made in describing the molecular and cellular changes that occur within spinal elements, while spinal deformity develops [9-15]. A thesis accounting for these dynamic changes, the 'vicious cycle' model [16,17], is discussed in this review in the context of clinical implications for prevention and control of spinal deformity.

**Table 1:** Research into Possible Cause and Effect in Spinal Deformity

1967. IS: An investigation of *genetic and environmental factors*. J Bone Jt Surg 49-A: 1005
1974. The early onset of *osteoarthritis *in juvenile and AIS. J Bone Jt Surg 56: 1575
1979. *Equilibrium factors *as predictors of the prognosis in AIS. Clin Orthop and Related Res 152: 232
1980. Analysis of *lateral predominance *in AIS with special reference to curve convexity. Spine 5: 512.
1981. *Proprioceptive function *in children in AIS. Spine 6: 560
1988. *Growth and ethnicity *in scoliosis. Acta Orthop Scand 59: 310
1988. *Growth hormone profiles *in pubertal girls with AIS. Spine 13: 139
1988. Idiopathic scoliosis: *clinical, morphometric, histopathological correlation*. J Pediatric Orth 8:147
1988. *Muscle spindles *in the paraspinal musculature of patients with AIS. Spine 13: 461
1989. *Equilibrial dysfunction *in scoliosis–cause or effect? J Spinal Disord 2: 184
1989. *Psychological implications *of genetic factors in scoliosis. Loss, Grief are 3: 169
1989. *Spinal mobility *in AIS and normal controls. Spine 14: 757
1990. *Zinc status *in patients with IS. Spine 15: 65
1991. A theory concerning prenatal origins of *cerebral lateralization *in humans. Psych Review 98: 299
1991. Hereditary *orthodontic anomalies *and IS. Int Orthop 15: 57
1991. Idiopathic scoliosis and *asymmetry of form and function*. Spine 16: 84
1991. MRI imaging of the *brain stem *in AIS. Spine 16: 761
1993. AIS and *joint laxity*: a study with somatosensory evoked potentials. Spine 18: 918
1993. AIS: *early menarche*, normal growth. Spine 18: 529–535
1993. *Complex balance reactions *in different sensory conditions: Adolescents +/- IS. J Orthop Res 11: 215
1994. The potential role of the *elastic fiber system *in AIS. J Bone Jt Surg 76-A: 1193
1995. Decreased incidence of scoliosis in *hearing impaired *children. Spine 20: 776
1995. *Increased femoral neck shaft angles *in AIS. Spine 20: 303
1996. *Melatonin*, possible role in pathogenesis of AIS. Spine 21: 1147
1997. *Abdominal reflexes*. J Pediatric Orthop 17: 105
1997. Incidence and risk factors for *mitral valve prolapse *in severe AIS. Pediatric Cardiol 18: 425
1997. *Osteopenia *in AIS: A primary problem or secondary to the spinal deformity? Spine 22: 1716
1998. *Neural axis abnormalities *in infantile and juvenile patients with spinal deformity. Spine 23: 206
1999. A *genomic approach *to scoliosis pathogenesis. Lupus 8:356
1999. *Familial back shape *in AIS. Acta Orthop Scand 62: 131
1999. MRI evaluation of *multifidus muscles *in AIS. Pediatr Radiol 29: 360
1999. Preoperative, postoperative pathologic changes for *paravertebral muscles *in IS. J China Med 28: 131
1999. *Spinal cord insults *in the prenatal, perinatal, and neonatal periods. Dev Med Child Neurol 41: 311
2000. Generalized low areal and volumetric *bone mineral density *in AIS. J Bone Min Res 15: 1587
2000. IS: Relation between the *vertebral canal and the vertebral bodies*. Spine 25: 1360
2001. Evolution of scoliosis in six children treated with *growth hormone*. J Ped Orthop 10: 197
2001. Influences of different types of progressive IS on static and dynamic *postural control*. Spine 26: 1052
2001. The pathogenesis of IS: *uncoupled neuro-osseous growth*? Eur Spine J 10: 473
2001. *Visual deficiency *and scoliosis. Spine 26: 48
2002. A genetic locus for AIS linked to *chromosome 19p13.3*. Am J Hum Genet 71: 401
2002. Assignment of a locus for autosomal dominant IS to *human chromosome 17p11*. Hum Genet 111:401
2002. Association between *estrogen receptor gene polymorphisms *and curve severity of IS. Spine 27: 2357
2002. *Cellularity of human annulus tissue*. Histopathology 41: 531
2002. *Premature termination mutations in FBN1*. Am J Hum Genet 223
2003. Allelic variants of *human melatonin 1A receptor *in patients with familial AIS. Spine 28: 2025
2003. Cold-induced sweating syndrome is caused by mutations in *CRLF1 gene*. Am J Hum Gen 72: 375
2003. IS as a presenting sign of *familial neurologic abnormalities*. Spine 28: 40
2005. Abnormal *peri-pubertal anthropometric measurements and growth pattern *in AIS. Spine 28: 2152

## Development of scoliosis

### Causes of scoliosis

Scoliosis, described by Galenus as a lateral (side-to-side) curvature of the spine, can develop in anyone, at any point in life from infancy through old age. In some individuals scoliosis progresses to a complex three-dimensional disorder deforming the entire thorax. The upright human posture requires continuous, precise and intricate coordination between the central nervous system (CNS) and a complex array of bone, muscle, cartilage and other soft tissue. Therefore any disease, injury or mutation that results in failure of assembly or deterioration of any component can result in development of scoliosis [18-20]. Examples include CNS injury resulting in paralysis or cerebral palsy, poliomyelitis, and damage to bone structure from osteoarthritis or rickets [21-23]. A disease, genetic defect, or CNS injury, however, is not required for a spinal curvature to develop. A leg length discrepancy, for example, whether it is caused by cancer, a traumatic injury, or a birth defect, is among the factors known to cause spinal curvature [24]. Pain, psychological distress, muscle spasm or injury to soft tissues in the back also can cause scoliosis ranging in magnitude from mild to severe [25]. In young children the flexibility of the immature spine means that simple posturing or clenching in response to a painful lesion can result in *'an alarming degree of scoliosis' *[25].

**Figure 1:**
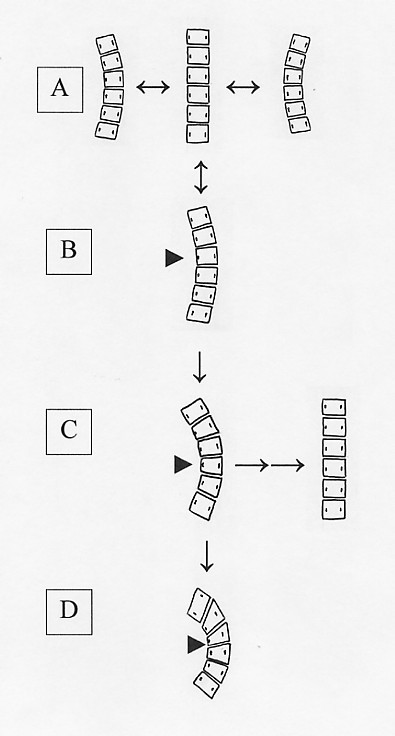
**Evolution of a structural deformity of the spine**. **A. Normal dynamics of spinal movement**. A normal human spine is programmed to assume a wide range of positions, including curvatures to the left or right ('scoliosis'), in response to stimuli. Such curvatures are transient and reversible and occur numerous times during the course of a day. **B. Functional curvature**. Radiographs of an individual with an asymmetric posture commonly reveal a spinal curvature which resolves when the patient adjusts his posture. Any curvature which reverts to a straight spine when the person bends to the side or lies down is considered to be a functional curvature, not a spinal deformity. As in a normal spine, the curvature, rotation of vertebrae, and associated torso imbalance are flexible and reversible, and there is no deformation of vertebral bodies (triangle). In some cases, such as a trauma-induced scoliosis due to a car wreck, the functional curvature may last for a few days and then resolve when the injuries heal and the pain resolves. In other cases, such as a pain-induced curvature in which the injury is never treated appropriately, the functional curvature may become habitual and linger indefinitely. In older children, once a curvature has been present for more than a year it usually will not resolve even if the inciting problem does resolve [19,29]. Eventually, a state of continuous asymmetric loading is established and maintained sufficiently to reach a threshold required to affect growth plates within the spinal bones. **C. Structural curvature: Before skeletal maturity**. Ultimately, under the constant stress of asymmetric loading, there is a predictable change in skeletal architecture (triangle), and the curvature evolves into a spinal deformity which no longer is flexible and readily reversible. Once this occurs, there is a fixed asymmetric deformity of the torso that does not resolve when the patient adjusts his posture. Once the curvature has progressed into a structural deformity, it still can be mild, nondeforming, and of little threat to the person's health and well-being. However, the vicious cycle model predicts that the continuous asymmetric load, however small, will push it in the direction of progression unless steps are taken to counteract it. The more asymmetric the load, the likelier it is that the curvature will progress. Yet, even with severe structural deformities the curvature can be reversed if the state of continuous loading is reversed and symmetrical pressure on the growth plates is restored (right). **D. Structural curvature: After skeletal maturity**. Once bone growth is complete, vertebral deformities persist for life. However, despite the structural deformity at the apex of the curvature, other parts of the spine remain flexible and can still correct on side bending [33,78,80–82]. Thus, a curvature measuring 50 degrees in the standing position may correct to 30 degrees in the supine position. This 20-degree 'functional' component of the curvature can still be corrected by a change in posture, but the overall flexibility of the spine decreases with age [78,79]. Progression of the curvature results from continued asymmetric loading of the deformed vertebral elements, at an average rate of 10 degrees per decade, with a corresponding loss in height of 1.5 cm per decade beginning in early adulthoold [83].

Most spinal deformities begin as a so-called 'nonstructural' or 'functional' scoliosis [25-27]. An exception is curvatures resulting from a congenital malformation of the spine; such congenital scolioses are not considered in this review. In the normal human spine, temporary reversible curvatures to one side or the other occur naturally as a response to an asymmetric posture (Figure 1A). Even when such a curvature becomes habitual it can remain reversible (Figure 1B). By definition, a functional curve resolves and the spine resumes a straight configuration when the patient lies down or bends to the side. A lateral spinal curvature which can be corrected completely by using a shoe lift to balance a leg length discrepancy is one example of a functional scoliosis [28]. Functional scoliosis develops in association with benign tumors and can resolve spontaneously within a year or two after the tumor regresses or is removed surgically [18,29]. Children and adolescents develop so-called 'hysterical' scoliosis in response to psychological distress [30,31]. Hysterical scoliosis clinically may be indistinguishable in appearance and magnitude from that caused by other factors, and the diagnosis has been applied incorrectly, for example, in curvatures that develop in response to bone tumors [32-34]. Yet, as with any functional scoliosis, the curvature straightens in response to bending sideways.

### Evolution from nonstructural to structural scoliosis

In contrast to a functional spinal curvature, a 'structural' scoliosis is associated with a loss of flexibility in one or more segments of the curved spinal column [20,28,32-34]. When the patient is radiographed while bending to the side, lying on his back (supine), or unconscious, the curvature is always present, even though its magnitude is reduced from that in a standing position (Figure 1C). At this point in the development of scoliosis, a structural spinal deformity is judged to be present.

It has been fashionable in recent decades to presume a separate etiology for nonstructural and structural spinal curvatures [28,32,34]. Nonstructural scoliosis is dismissed as an inconsequential and largely benign effect of 'bad posture,' posture being defined by the AMA as *'The relative position of different parts of the body at rest or during movement*' [35]. Structural scoliosis, in contrast, is seen as a genetically based disorder whose outcome largely is impervious to environmental influences [36]. Leatherman and Dickson [28] claim that structural scoliosis results from an *'inherent abnormality of the vertebral column or its supporting mechanisms' *and therefore has intrinsically more potential for progression than nonstructural scoliosis.

In truth, nonstructural scoliosis resulting from postural imbalance due to pain, muscle spasms, or other factors may progress over time into structural scoliosis if the inciting factors are not identified and corrected [37]. In early stages of scoliosis associated with leg length discrepancy, for example, such curvatures can be corrected by using a shoe lift to reduce the leg length discrepancy-associated postural asymmetry [32]. In established cases of spinal deformity occurring in correlation with leg length discrepancy, however, curvature magnitude can be reduced, but not corrected, by using a shoe lift [38,39]. That nonstructural scoliosis can develop into structural scoliosis was demonstrated by Paul Harrington [40], who induced postural imbalance by restricting movement in healthy inbred mice and compared the results with isogenic control populations. The results confirmed that postural imbalance, by itself, can cause severe structural scoliosis with vertebral rotation as well as wedging of vertebrae and intervertebral discs. The evolution of untreated nonstructural scoliosis into a fixed structural spinal deformity in children was described by Hipps [41], who identified young children with mild torso asymmetries. Supine radiographs revealed a straight spine, but in the standing position slight curvatures were present; this defines the children as having nonstructural scoliosis. Over the course of ten years, the curvatures progressed to structural deformities.

### Reversibility of structural scoliosis

Even after a spinal curvature has evolved into a spinal deformity, it may still be reversed if the postural asymmetry is removed while significant growth potential remains (Figure 1C). Harrington [40] reported that severe structural curvatures induced by postural asymmetry in mouse resolved completely when the postural imbalance was removed. A similar phenomenon occurs in pain-provoked scoliosis in children when the underlying cause of the postural imbalance is quickly diagnosed and treated [29]. In two cases, for example, children developed pain-provoked scoliosis in response to tumors that healed within a year and the associated spinal curvatures resolved within a year after that [19]. But three children were misdiagnosed for two, three, and six years, respectively, and when the painful lesion finally healed the scoliosis did not. Instead, the curvatures progressed to moderate and severe fixed deformities with Cobb angles ranging from 42 to 62 degrees by early adolescence. After skeletal maturity, resolution of spinal deformity has not been reported to occur; some cases, in fact, continue to worsen throughout life (Figure 1D).

## Diagnosis and clinical consequences

Like many other chronic diseases, scoliosis may be present and asymptomatic for months or years before it becomes sufficiently severe to be detected. Before the advent of school screening programs in the 1970s and 1980s, few cases were diagnosed before they were moderate or severe deformities [28]. Even when screening programs are in place and more curvatures are detected while they are still mild, by the time scoliosis finally is diagnosed the cause of the scoliosis is no longer apparent in most cases. Therefore, most scoliosis is classified by default as being 'of unknown origin' or 'idiopathic.' In idiopathic scoliosis (IS) the patient is healthy except for the presence of the spinal curvature whose cause is not identified [21,32,33]. For 70–80% of IS populations, there is no evidence for an inherited susceptibility among family members, and the curvature presumably is due to an undiagnosed injury or disease process that may have resolved earlier in life [42]. For 20–30% of IS patient populations, one or more members of the immediate family also have scoliosis, suggesting that an inherited factor plays a role [43]. In such familial IS, the mechanism that triggers a spinal curvature might be fundamentally distinct from that of other patients. Alternatively, familial IS may involve a predisposition to develop scoliosis in response to the same factors that can cause it in anyone.

Irrespective of the factor that triggered its development, once a structural deformity is present, the pathological consequences among populations of scoliosis patient share common elements. These elements include a progressive loss of torso mobility resulting from the fixed postural asymmetry, and a consequent reduction in chest wall movement and vital capacity [44]. Pain in populations of young adult scoliosis patients, irrespective of curvature magnitude, is increased compared with control populations [45]. At > 44-year follow-up of a group of patients diagnosed in adolescence, incidence of pain was double that of a group of similar age without scoliosis [46]. This is despite the fact that the 'control' population for the study was selected from hospital clinics, nursing homes, and senior citizens centers where incidence of disability is exceptionally high [47]. Every patient with a structural scoliosis present at skeletal maturity potentially faces a lifelong disease burden. The younger the child at the time the structural deformity develops, the more severe the symptoms, and scoliosis developing in infancy brings high risk of serious complications including respiratory failure [48]. Central to the transformation of a reversible spinal curvature into a structural spinal deformity, irrespective of the factor(s) that trigger its development, is a characteristic wedge-shaped deformity of the vertebral bodies that appears early in the disease process [49]. This vertebral deformity sets the stage for a 'vicious cycle' of curvature progression and symptom development [reviewed in [16,17]].

## Pathological changes in structure and function in response to asymmetric loading: cause and effect

The bony axis of the human spine, which by itself cannot tolerate a weight of > 10 kilograms without buckling, depends for stability on a balanced muscular system coordinated by the CNS [50]. The effects of gravity on the upright human posture are powerful: Individuals are as much as 25 mm taller in the morning than in the evening, as a result of compressive forces bearing down all day [51,52], and astronauts 'grow' by nearly 75 mm when released from the force of the earth's gravity [53]. In spinal deformity, the same forces are bearing down on a curved spinal column without balanced support from the musculoskeletal system. Roaf [16] proposed that asymmetric loading of the vertebral axis is the primary driving force for the development and progression of a spinal deformity: Once a curvature develops, unequal compression on vertebral plates results in unequal growth, which in turn contributes to the progression of the deformity. Asymmetrical changes in rib and vertebral structure and function predictably follow from the asymmetric stresses applied in a spinal curvature [54,55]. For any kind of machinery from a misaligned automobile to a human spine, asymmetrical loading constitutes a 'vicious cycle' which tends to perpetuate itself: The more unbalanced the load, the more likely it will become even more unbalanced over time under the relentless influence of gravity.

The model predicts that once a spinal curvature is triggered and continuous asymmetric loading is established, mechanical forces imposed by asymmetric loading directly cause structures of the spinal column to become deformed [17]. Such deformities in turn create a new level of fixed asymmetric loading that leads to continued progression. Thus, the vicious cycle defines a paradigm in which fixed asymmetric spinal loading is cause AND effect, and explains why the danger of progression is so high in patients during periods of rapid growth: asymmetric loading actually inhibits growth within affected spinal elements.

## Molecular, cellular, and clinical predictions of the 'Vicious Cycle' model

The 'vicious cycle' model is of value for its potential to bridge basic science and clinical applications by generating predictions that can be quantified in the laboratory, in individuals over time, and among patient populations [56]. Research to explore this hypothesis has addressed the fundamental questions of how the spine is loaded when scoliosis is present, how growth responds to this altered load, and how much of scoliosis progression in the coronal ('frontal') plane can be attributed to mechanically modulated growth [57-62]. The results, summarized below, support the premise that lateral spinal curvature results in asymmetric loading which, in turn, affects gene expression underlying the structure and function of growth plates within the spine [9-15]. These changes, in turn, foster the development and progression of scoliosis. An equal balance of compression on growth plates of a symmetrically loaded vertebral column yields a straight spine. Unrelieved contrasting forces on each of the two sides of a vertebral growth plate, however, quickly produce within vertebrae and intervertebral discs a wedged deformity whose magnitude can account for most if not all of the lateral curvature that develops in a progressive scoliosis [58,59,61,62]. Even spinal curvatures due to CNS injury in infancy may remain stable throughout most of childhood, but worsen markedly during the period of rapid growth at adolescence [63,64]. Differences in progression among individual patients may stem from divergence in muscle activation strategies rather than an inherent deficiency in structure and function within the spine [61]. Such differences in muscle activation strategies might also explain the observation that simple 'side shift' exercises were correlated with curvature stabilization in two groups of patients at high risk of progression, by transient repeated reversal of asymmetric loading [65,66]. Continuous steady state loading inhibits growth but transient loading apparently does not [17].

The vicious cycle model predicts that, once asymmetric loading is established and maintained beyond a critical threshold for weight and time, there will be an inevitable tendency for progression to occur unless compensatory action offsets the biomechanical effects of the imbalance. Most important, when the load asymmetry is removed while significant growth potential remains, progression stops; when the asymmetry of the vertebral column is reversed and the unbalanced loading is thereby corrected, complete resolution of deformity occurs [19,40]. The model explains why spinal deformities in children and in experimental animals can resolve, when the inciting cause of postural asymmetry is reversed, because vertebral growth is not permanently affected by applied loading [58]. Reversing the asymmetric loading by restoring normal posture and movement therefore allows even severe structural curvatures to resolve completely.

## Transition from spinal curvature to spinal deformity: A molecular mechanism?

Several recent articles have reported structural and functional changes consistent with predictions of the vicious cycle model, and suggesting a possible molecular mechanism by which progression occurs. Parent et al., [67] compared the pathological consequences of scoliosis on each vertebra within each of thirty human spines, with thirty control specimens from individuals without scoliosis. The samples were matched for age, sex, race, height and weight. The results revealed that vertebral wedging was consistent among the population, occurred mainly at the apex of thoracic curves and was primarily in the coronal plane. There was no deformity in the sagittal plane. This uniformity of structural transformation would be the expected result if progression among all individuals resulted from discrepancy in growth at the vertebral plates, due to unequal side-to-side loading. Using a different approach, Villemure et al., [14] found a similar pattern of deformity. Among 28 adolescents whose deformities were measured over time, as they developed, there was a consistent pattern for lateral wedging of vertebral elements as would be predicted if their evolution shared a common mechanism [14]. There was no significant correlation among the group for progression in the sagittal plane.

Several groups have documented changes in gene expression in intervertebral tissues of scoliosis patients, compared with control subjects [8-15]. A study of intervertebral discs and endplates revealed a possible molecular mechanism by which growth is altered by mechanical loading: subjects with scoliosis exhibited an apparent inhibition of matrix turnover attributed to the 'pathological mechanical environment' [9]. Altered matrix turnover occurring in response to continuous asymmetric loading could account for observations that increased cell death occurs in discs of patients with scoliosis [68]. Mechanical stress has been implicated in the activation of programmed cell death ('apoptosis') in human somatic tissues [69-72]. In mouse intervertebral discs, continuous compression loads of 1.0 MPa result in onset of programmed cell death within 24 h [73]. The number of apoptotic cells increased with increased load; there was no apoptosis in discs that were not subjected to mechanical stress.

Programmed cell death within intervertebral discs of a group of sixteen surgery patients with idiopathic or neuromuscular scoliosis, aged 10–17 or 17–48 years, respectively, was examined [10]. Cell death was highest within cells at the apex of the curvature, where mechanical loading is highest, and was similar for all age groups and for subjects with neuromuscular or idiopathic scoliosis. This result suggests that the observed changes occurred via a common pathway for pathogenesis despite divergent histories, stages of growth, and triggers for initiation of scoliosis. It is reasonable to predict that the activation of programmed cell death in response to mechanical loading comprises the molecular mechanism by which a reversible spinal curvature is converted into an irreversible spinal deformity. Programmed cell death in response to a threshold of mechanical loading might also account for the observation that spinal deformities can continue to increase in magnitude in adults, after growth is complete.

## Progression of spinal deformity in adults

Deformities present at skeletal maturity persist for life and can continue to progress over time [74-79]. The mechanism for progression of scoliosis in adults is not well defined but presumably involves remodelling of tissues by 'wear-and-tear' effects of continuous loading, since growth potential is absent. Adult curvatures repeatedly have been found to progress in proportion to curvature magnitude [74-79]. This observation is consistent with the possibility that, in adults as well as in children, progression results from biomechanical loading imbalance and therefore increased loading fosters increased progression. Thus, in one study of 187 patients followed for > 15 years after skeletal maturity, 20–29 degree curvatures progressed 10 degrees, on average; 30–39 degree curvatures progressed 12 degrees; 40–49 degree curvatures progressed 15 degrees; and 50–59 degree curvatures progressed 20 degrees [74]. As in children, variation in progression among adult patients with similar curvatures may be predicted to result from different muscle activation strategies that alter the loading imbalance. Curvatures of less than 20 degrees are less likely to progress than more severe curves, perhaps because they produce mechanical loads below the threshold required to induce cellular changes leading to degenerative changes in spinal elements. However, even mild curves that remain stable become increasingly rigid with age and are associated with reduced pulmonary function and increased pain that result, presumably, from secondary effects of altered mechanical loading [45,46,76-79].

## Conclusion

A significant body of research now has demonstrated that, whatever the initial trigger that induces a spinal curvature, asymmetric loading of the spinal axis produces biomechanical forces that can account for most if not all progression of the spinal deformity [9-17,57-62,80]. The data, taken together, suggest that there is a threshold for continous asymmetric loading that must be reached before vertebral changes occur, and that transient loading will not foster asymmetric growth leading to deformity. Muscle activation strategies that offset the loading can be predicted to account for patient-specific differences in evolution of a functional curvature into a progressive structural scoliosis [14,61]. Structural damage to bone and disc can occur very early in the development of even minor curves [49]. Yet the damage can be reversed entirely if steps are taken to reverse the loading imbalance while significant growth potential remains [19,40,58]. These data suggest that preventing a state of continuous asymmetric loading in children in early stages of scoliosis will prevent the development of spinal deformities. Continued research to develop methods to quantify the status of spinal loading in individual patients, and thereby define its potential for causing curvature progression, is of paramount importance [57-62,78,81-85]. In the meantime, sufficient data in support of the 'vicious cycle' model are available to justify empirical studies to explore the use of simple daily exercises or other interventions, such as those described by Maruyama and co-workers [65] and by Mehta [66]. Such exercises, designed to interrupt steady state spinal loading at the apex of the curvature, can be predicted to forestall the cascade of molecular events that transform benign spinal curvatures into progressive spinal deformities.

## Competing interests

The author(s) declare that they have no competing interests.

## Authors' contributions

Both authors contributed materially to the concepts presented herein, and to the writing of the manuscript. The first author carried out the literature review.
